# Severity of thinness amplifies mortality risk in patients with transcatheter aortic valve implantation

**DOI:** 10.1093/icvts/ivaf120

**Published:** 2025-06-20

**Authors:** Kazuma Handa, Koichi Maeda, Kyongsun Pak, Kazuo Shimamura, Koichi Inoue, Shohei Yamada, Kizuku Yamashita, Ai Kawamura, Daisuke Yoshioka, Shigeru Miyagawa

**Affiliations:** Department of Cardiovascular Surgery, Osaka University Graduate School of Medicine, Suita City, Osaka, Japan; Department of Cardiovascular Surgery, Osaka University Graduate School of Medicine, Suita City, Osaka, Japan; Division of Biostatistics, Center for Clinical Research, National Center for Child Health and Development, Setagaya, Japan; Department of Cardiovascular Surgery, Osaka University Graduate School of Medicine, Suita City, Osaka, Japan; Department of Cardiovascular Surgery, Osaka University Graduate School of Medicine, Suita City, Osaka, Japan; Department of Cardiovascular Surgery, Osaka University Graduate School of Medicine, Suita City, Osaka, Japan; Department of Cardiovascular Surgery, Osaka University Graduate School of Medicine, Suita City, Osaka, Japan; Department of Cardiovascular Surgery, Osaka University Graduate School of Medicine, Suita City, Osaka, Japan; Department of Cardiovascular Surgery, Osaka University Graduate School of Medicine, Suita City, Osaka, Japan; Department of Cardiovascular Surgery, Osaka University Graduate School of Medicine, Suita City, Osaka, Japan

**Keywords:** aortic stenosis, transcatheter aortic valve implantation, underweight, thinness

## Abstract

**OBJECTIVES:**

Underweight (body mass index [BMI]<18.5 kg/m^2^), which is categorized into mild, moderate and severe thinness, increases mortality risk in patients undergoing transcatheter aortic valve implantation (TAVI). However, the impact of the severity of thinness, especially of the severe thinness, on mortality after TAVI remains unclear.

**METHODS:**

A total of 1804 consecutive patients with TAVI from the Japanese multicentre aortic stenosis registry were included. Of these, 60 (3.3%) patients with severe thinness, 213 (11.8%) with mild–moderate thinness and 1161 (64.4%) with normal weight were assessed for in-hospital and mid-term outcomes.

**RESULTS:**

While there were no differences in age, male sex, malignancy or ejection fraction among the groups, the severe thinness group exhibited lower albumin and haemoglobin levels. The severe thinness group had higher in-hospital mortality (6.7% vs 0.0% vs 0.7%, *P *= 0.001). Median follow-up period was 4.3 [3.8–5.0] years. Cumulative all-cause mortality at 5 years was significantly higher in the severe thinness than in the mild–moderate thinness and normal-weight groups (77.9% vs 61.6% vs 44.4%, *P *< 0.001). The severe thinness group had a higher cumulative incidence of not only cardiac mortality (50.2% vs 35.0% vs 26.5%, *P *= 0.002) but also non-cardiac mortality (55.7% vs 40.9% vs 28.6%, *P *< 0.001). Multivariable analysis demonstrated that severe thinness (adjusted hazard ratio [aHR], 3.00; 95%confidence interval [CI], 2.03–4.41, *P *< 0.001) and mild–moderate thinness (aHR, 1.35; 95% CI, 1.03–1.76, *P *= 0.027) were independent risk factors for the mid-term mortality.

**CONCLUSIONS:**

Severe thinness increases the risk of in-hospital mortality. Moreover, the severity of thinness strongly amplifies mid-term mortality after TAVI.

## GRAPHICAL ABSTRACT

**Figure:**
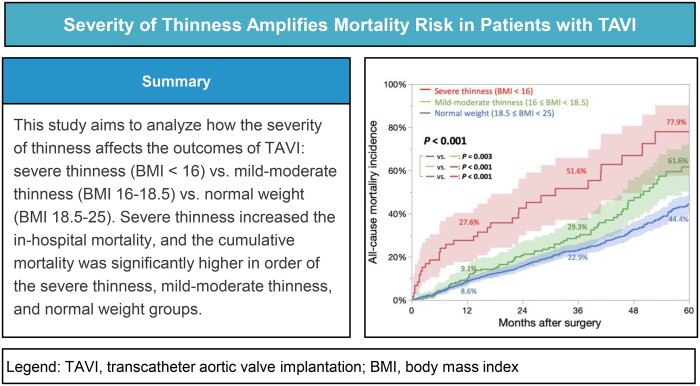

## INTRODUCTION

A global increase in the older adult population has led to an increase in the prevalence of aortic stenosis (AS) [1]. Transcatheter aortic valve implantation (TAVI) has been recommended in patients above 75 years or in those who are high risk for surgical aortic valve replacement (SAVR) [2–4]. Consequently, the number of TAVI procedures has rapidly increased worldwide, and it is now performed more frequently than SAVR in both Western countries and East Asia [5].

Obesity is associated with a better prognosis [6]. However, being underweight—caused by various conditions such as malnutrition, metabolic and endocrine disorders, malignancies and heart failure—is more common among patients undergoing TAVI in East Asia but less prevalent in Western countries [7]. Notably, underweight status is associated with a worse prognosis compared to normal weight [8]. According to the World Health Organization (WHO) classification, underweight is further categorized as mild, moderate and severe thinness [9]. However, little is known about whether the severity of thinness affects the prognosis of underweight patients undergoing TAVI. We designed this study based on the hypothesis that severe thinness could disproportionately worsen overall mortality in underweight TAVI patients. In this study, we aimed to analyse how the severity of thinness, especially the severe thinness, affects mid-term mortality in patients with TAVI using data from a multicenter Japanese registry, ACTIVIST (Accelerating Conventional and Transcatheter Integration in Valvular Intervention STrategy).

## MATERIALS AND METHODS

### Study population

The ACTIVIST registry is a retrospective multicenter registry that includes all patients who undergo isolated SAVR or TAVI for severe AS at five centers in Japan. These centers are accredited as TAVI facilities in Japan after a rigorous selection process, and the decision regarding the procedure is made by a team of cardiovascular surgeons and cardiologists based on Japanese, American and European guidelines [2–4]. As the registry is based on the Japan Adult Cardiovascular Surgery Database, the definitions of these comorbidities can be found online at http://www.jacvsd.umin.jp.

Between January 2016 and December 2021, 1804 consecutive patients with TAVI were registered at these institutions. Height and weight were measured after admission and prior to surgery, and body mass index (BMI) was classified according to the WHO criteria [9]. Among the 1434 patients remaining after excluding 370 obesity (BMI ≥25 kg/m^2^) patients (20.5%), we analysed patient backgrounds, surgical data and in-hospital and mid-term outcomes among three groups: severe thinness (BMI <16 kg/m^2^), mild–moderate thinness (BMI 16–18.5 kg/m^2^) and normal weight (BMI 18.5–25 kg/m^2^).

Echocardiographic data were collected at three time points: (i) baseline before surgery, (ii) at discharge following TAVI and (iii) 1-year post-surgery. After discharge, outpatient follow-up was performed every 6–12 months. Patients who could not attend outpatient visits were followed-up by telephone as needed.

### Ethical statement

This study was conducted in accordance with the principles of the Declaration of Helsinki. The Clinical Research Ethics Committee of Osaka University Hospital approved this study and published the data (Approval No.: 20222 [T2]; Approval Date: December 15, 2021). Written informed consent was obtained from all the participants.

### Endpoints

All clinical events were recorded and defined according to the criteria established by the Valve Academic Research Consortium-3 [10]. In-hospital outcomes, including operative data, permanent pacemaker implantation, stroke, length of hospitalization, paravalvular leak (PVL) ≥ moderate, all-cause mortality, and cardiac and non-cardiac mortality, were evaluated. For mid-term outcomes up to 5 years, we compared all-cause, cardiac and non-cardiac mortality among the three groups.

### Statistical analysis

Categorical variables are presented as frequencies and percentages (%), and continuous variables are presented as medians and interquartile ranges. The Fisher’s exact test was used to compare categorical variables between independent groups. The Kruskal–Wallis test was used to compare continuous variables among the three groups. The cumulative follow-up period and cumulative incidence were calculated using the inverse Kaplan–Meier method and compared using the log-rank test. To estimate the risk of all-cause mortality in each BMI group with normal weight as a reference, a multivariable Cox proportional hazards model was developed. The model was adjusted for preoperative and surgical (elective surgery, transfemoral [TF] approach, prosthesis-patient mismatch [PPM] ≥ moderate and postoperative PVL ≥ moderate) factors identified as potential confounders in univariable analysis (*P *< 0.1), in addition to age and sex. Statistical significance was set at *P *< 0.05. All statistical analyses were performed using JMP Pro (version 17.1.0, SAS Institute, Inc.).

## RESULTS

### Study population

**Figure 1:**
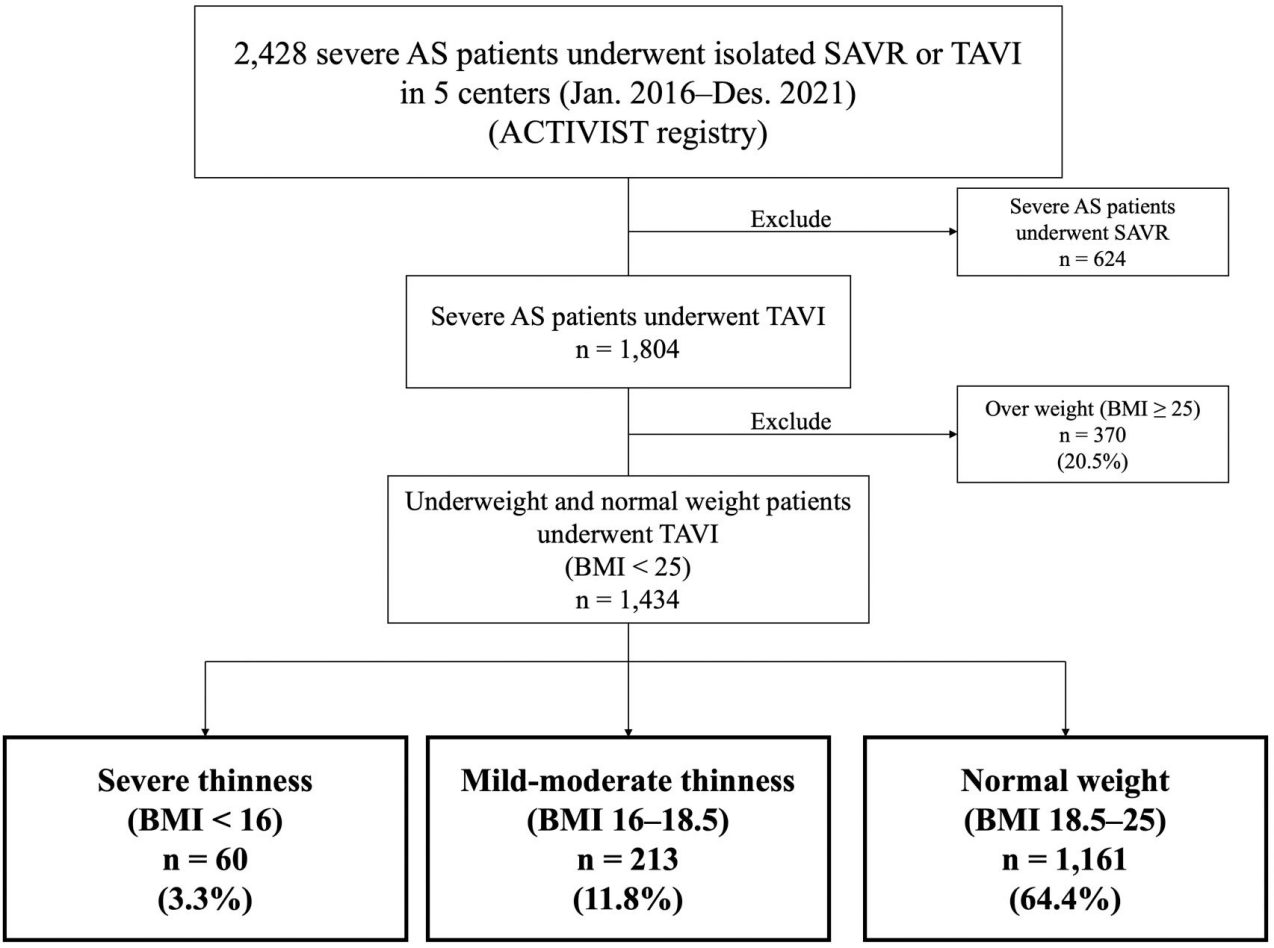
Study flowchart. AS: aortic stenosis; TAVI: transcatheter aortic valve replacement; BMI: body mass index

The included 1434 patients were classified into three groups: 60 (3.3%) patients with severe thinness (BMI <16 kg/m^2^), 213 (11.8%) with mild–moderate thinness (BMI 16–18.5 kg/m^2^) and 1161 (64.4%) with normal weight (BMI 18.5–25 kg/m^2^) (Fig. 1). The patient backgrounds in each group are shown in Table 1. There were no significant differences in age (85 [80–89] vs 85 [82–88] vs 85 [81–88] years, *P *= 0.626), male sex (68.3% vs 59.2% vs 58.2%, *P *= 0.298) or malignancy (6.7% vs 10.8% vs 7.6%, *P *= 0.262) between the severe thinness, mild–moderate thinness and normal weight groups. Serum albumin (3.4 [3.0–3.8] g/dl vs 3.6 [3.3–3.9] g/dl vs 3.7 [3.4–4.0] g/dl, *P *< 0.001) and hemoglobin (10.4 [9.25–11.4] g/dl vs 11.2 [9.7–12.3] g/dl vs 11.3 [10.2–12.4] g/dl, *P *< 0.001) levels were significantly lower in the severe thinness group than those in the other groups, and liver cirrhosis was more prevalent in the severe thinness group (6.7% vs 0.9% vs 1.0%, *P *= 0.009). No significant difference was observed in left ventricular ejection fraction (LVEF) (64.0 [55.5–73.0]% vs 64.0 [53.0–72.0]% vs 65.0 [57.7–71.0]%, *P *= 0.541) or the Society of Thoracic Surgeons Predicted Risk of Mortality (STS-PROM) (5.65 [3.53–8.26]% vs 5.60 [4.19–9.52]% vs 5.59 [4.07–8.28]%, *P *= 0.301) among the three groups.

**Table 1:** Baseline characteristics

Variable	Severe thinness (*n* = 60)	Mild–moderate thinness (*n* = 213)	Normal weight (*n* = 1161)	*P*-value
Age (years)	85 [80–89]	85 [82–88]	85 [81–88]	0.626
Male sex	41 (68.3)	126 (59.2)	676 (58.2)	0.298
DM	6 (10.0)	46 (21.6)	333 (28.7)	**0.001**
eGFR (ml/min/1.73 m^2^)	52.4 [36.5–73.3]	51.9 [36.4–64.8]	49.0 [35.9–61.3]	0.167
Haemodialysis	4 (6.7)	14 (6.6)	66 (5.7)	0.847
Albumin (g/dl)	3.4 [3.0–3.8]	3.6 [3.3–3.9]	3.7 [3.4–4.0]	**<0.001**
Haemoglobin (g/dl)	10.4 [9.25–11.4]	11.2 [9.7–12.3]	11.3 [10.2–12.4]	**<0.001**
Hypertension	37 (61.7)	150 (70.4)	863 (74.3)	0.059
Active IE	0 (0)	0 (0)	0 (0)	>0.999
COPD ≥ moderate	8 (13.3)	18 (8.5)	118 (10.2)	0.513
Immunosuppressant drugs	5 (8.3)	22 (10.3)	104 (9.0)	0.796
Peripheral artery disease	5 (8.3)	22 (10.3)	131 (11.3)	0.731
Cerebrovascular disease	5 (8.3)	30 (14.1)	137 (11.8)	0.431
Malignant disease	4 (6.7)	23 (10.8)	88 (7.6)	0.262
Liver cirrhosis (child ≥ B)	4 (6.7)	2 (0.9)	12 (1.0)	**0.009**
Coronary artery disease	8 (13.3)	45 (21.1)	278 (23.1)	0.125
NYHA ≥III	29 (48.3)	82 (38.5)	356 (30.7)	**0.003**
Atrial fibrillation	16 (26.7)	34 (16.0)	188 (16.2)	0.101
LVEF (%)	64.0 [55.5–73.0]	64.0 [53.0–72.0]	65.0 [57.7–71.0]	0.541
EF < 50%	9 (15.0)	47 (22.1)	180 (15.5)	0.067
Mean PG (mmHg)	39.3 [27.1–49.5]	44.4 [37.0–54.2]	45.0 [37.0–56.0]	**0.001**
LV mass index (g/m^2^)	120.7 [84.8–142.2]	122.7 [101.2–144.4]	125.0 [103.5–148.4]	0.149
STS-PROM (%)	5.65 [3.53–8.26]	5.60 [4.19–9.52]	5.59 [4.07–8.28]	0.301

Data are expressed as *n* (%) or median [interquartile range]. Values in bold indicate statistically significant. STS: Society of Thoracic Surgeons; DM: diabetes mellitus; eGFR: estimated glomerular filtration rate; IE: infective endocarditis; COPD: chronic obstructive pulmonary disease; NYHA: New York Heart Association; LVEF: left ventricular ejection fraction; PG: pressure gradient; LV: left ventricular; STS-PROM: Society of Thoracic Surgeons Predicted Risk of Mortality.

### Operative data and in-hospital outcomes

Operative data and in-hospital outcomes are shown in Table 2. There were no significant differences in the use of the TF approach (93.3% vs 93.4% vs 93.5%, *P *= 0.996) or type of prosthetic valve (self-expandable: 38.3% vs 43.9% vs 42.5%, *P *= 0.793) among the severe thinness, mild–moderate thinness and normal weight groups, respectively. Regarding in-hospital outcomes, the severe thinness group exhibited a significantly longer postoperative hospitalization period (10 [6.3–19.8] days vs 8 [6–12] days vs 8 [6–12] days, *P *= 0.008) and a significantly higher rate of all-cause mortality (6.7% [4/60] vs 0.0% [0/213] vs 0.7% [8/1161], *P *= 0.001) than those in the other groups. There were no significant differences in the cardiac mortality rates among the three groups (1.7% [1/60] vs 0.0% [0/213] vs 0.3% [3/1161], *P *= 0.255). However, non-cardiac mortality was significantly higher in the severe thinness group (5.0% [3/60] vs 0.0% [0/213] vs 0.3% [5/1161], *P *= 0.001), with 50% of these deaths attributed to respiratory diseases (Supplementary Table S1). Discharge echocardiography was not significantly different in LVEF (66.0 [59.0–72.6]% vs 66.0 [55.0–71.0]% vs 66.0 [59.0–71.0]%, *P *= 0.374) or the incidence of PVL ≥ moderate (3.6% vs 4.7% vs 3.4%, *P *= 0.554). However, the incidence of PPM ≥ moderate was significantly lower in the severe thinness group (3.6% vs 11.1% vs 13.1%, *P *= 0.044) than in the other groups (Table 2). At 1-year of follow-up, there were no significant differences in LVEF, PVL incidence, or PPM incidence (Supplementary Table S2).

**Table 2:** Operative data and in-hospital outcomes

Variable	Severe thinness (*n* = 60)	Mild–moderate thinness (*n* = 213)	Normal weight (*n* = 1161)	*P*-value
Operative data				
Elective	56 (93.3)	204 (95.2)	1111 (95.7)	0.679
Operative time (min)	72 [58–103]	70 [60–89]	70 [59–88]	0.873
TF-approach	56 (93.3)	199 (93.4)	1086 (93.5)	0.996
Prosthetic valve data				
Self-expandable	23 (38.3)	92 (43.9)	493 (42.5)	0.793
Valve size (mm)	26 [23–26]	26 [23–29]	26 [26–29]	**0.004**
Balloon-expandable	37 (61.7)	121 (56.1)	668 (57.5)	0.793
Valve size (mm)	23 [22.4–23]	23 [23–26]	23 [23–26]	0.185
In hospital outcomes				
PPI	9 (15.0)	18 (8.5)	93 (8.0)	0.162
Stroke	0 (0.0)	1 (0.5)	21 (1.8)	0.211
Hospitalization (days)	10 [6.3–19.8]	8 [6–12]	8 [6–12]	**0.008**
All-cause mortality	4 (6.7)	0 (0.0)	8 (0.7)	**0.001**
Non-cardiac mortality	3 (5.0)	0 (0.0)	5 (0.4)	**0.004**
Cardiac mortality	1 (1.7)	0 (0.0)	3 (0.3)	0.255
Discharge echocardiogram				
LVEF (%)	66.0 [59.0–72.6]	66.0 [55.0–71.0]	66.0 [59.0–71.0]	0.374
mean PG (mmHg)	7.9 [5.0–11.5]	8.0 [6.0–11.0]	9.0 [7.0–12.0]	**0.011**
LV mass index (g/m^2^)	113.8 [78.7–128.1]	112.9 [94.4–140.6]	119.1 [97.8–141.6]	**0.022**
PPM ≥ moderate	2 (3.6)	23 (11.1)	146 (13.1)	**0.044**
PVL grade ≥ moderate	2 (3.6)	10 (4.7)	40 (3.4)	0.554
PVL grade ≥ mild	22 (45.8)	88 (55.4)	509 (57.3)	0.279

Data are expressed as *n* (%) or median [interquartile range]. Values in bold indicate statistically significant. TF: transfemoral; PPI: permanent pacemaker implant; LVEF: left ventricular ejection fraction; PG: pressure gradient; LV: left ventricular; PPM: prosthesis-patient mismatch; PVL: paravalvular leak.

### Mid-term outcomes

**Figure 2:**
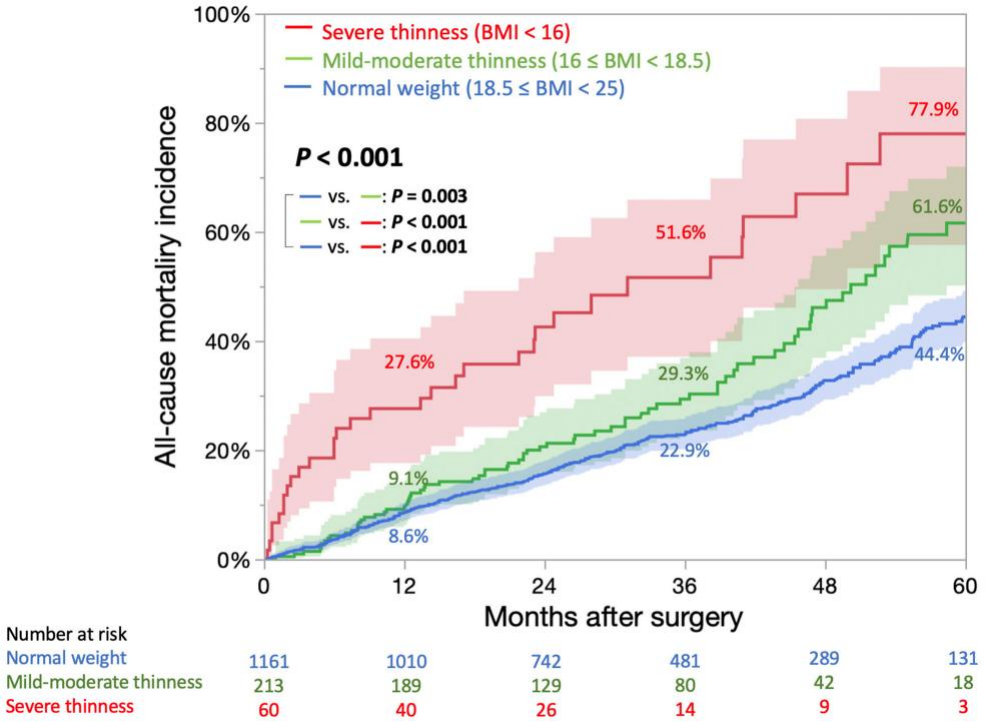
Kaplan–Meier curves for cumulative incidence of all-cause mortality. BMI: body mass index

**Figure 3:**
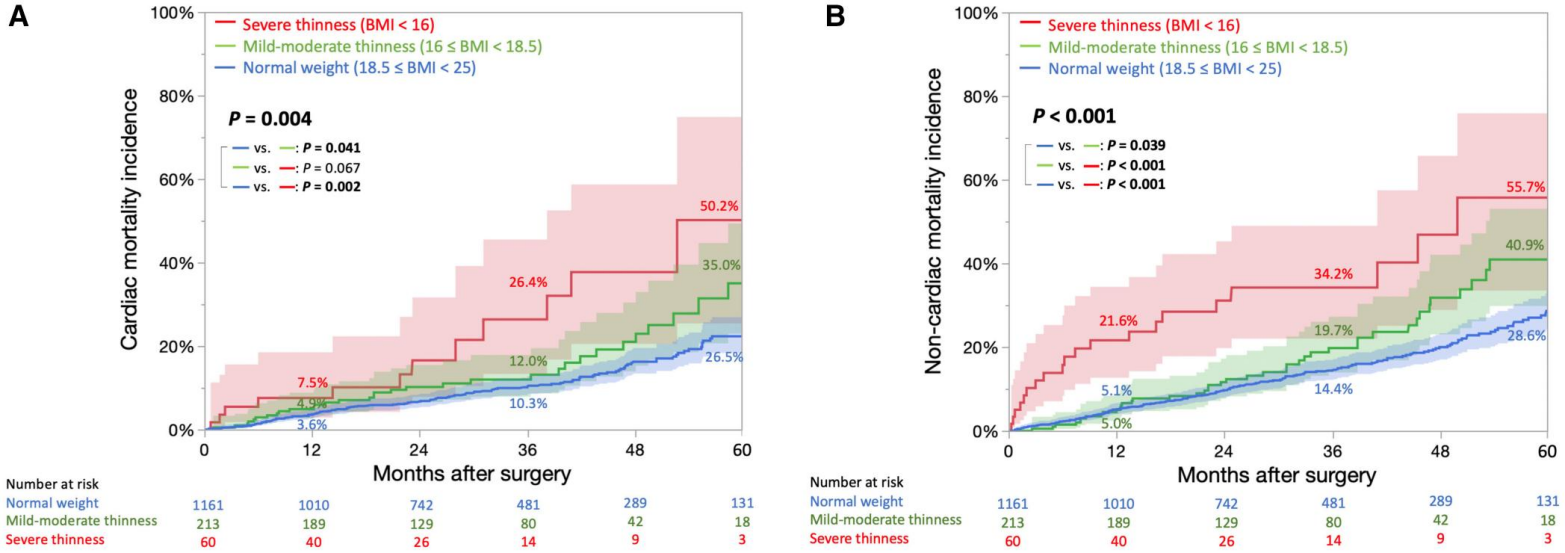
Kaplan–Meier curves for cumulative incidence of cardiac mortality (**A**) and non-cardiac mortality (**B**). BMI: body mass index

The median follow-up period was 4.3 [3.8–5.0] years. The underweight group (BMI < 18.5) had a significantly higher incidence of cumulative all-cause, cardiac and non-cardiac mortality than the normal weight group (Supplementary Fig. S1). During the follow-up, the number of mortalities was as follows: 296 of 1161 patients (25.5%) in the normal weight group (cardiac deaths, 39.9%; non-cardiac deaths, 60.1%); 74 of 213 (34.7%) in the mild–moderate thinness group (cardiac deaths, 43.2%; non-cardiac deaths, 56.8%) and 32 of 60 patients (53.3%) in the severe thinness group (cardiac deaths, 37.5%; non-cardiac deaths, 62.5%) (Supplementary Fig. S2). In the severe thinness group, a higher proportion of deaths was due to gastrointestinal and respiratory diseases, including pneumonia (Supplementary Table S3). The cumulative incidence curves of all-cause mortality were overall statistically significantly different with a *P*-value of <0.001, and 5-year cumulative all-cause mortality rates were 77.9%, 61.6% and 44.4% in the severe thinness, mild–moderate thinness and normal weight groups, respectively (Fig. 2, and Supplementary Fig. S3A). And the cumulative survival rates, including all events during hospitalization, were 22.1%, 38.4% and 55.6%, respectively. The severe thinness group had a higher cumulative incidence of not only cardiac mortality (50.2% vs 35.0% vs 26.5%; Fig. 3A, and Supplementary Fig. S3B) but also non-cardiac mortality (55.7% vs 40.9% vs 28.6%; Fig. 3B, and Supplementary Fig. S3C) than the mild–moderate thinness and normal weight groups at 5 years. Similar results were observed after excluding patients with liver cirrhosis (Supplementary Fig. S4).

### Risk analysis of BMI for 5-year follow-up mortality

In the univariable analysis, in addition to the severity of thinness, male sex, diabetes mellitus, estimated glomerular filtration rate, hemodialysis, albumin, hemoglobin, hypertension, chronic obstructive pulmonary disease, immunosuppressant drugs, peripheral artery disease, cerebrovascular disease, malignant disease, liver cirrhosis, New York Heart Association (NYHA), atrial fibrillation, LVEF, mean pressure gradient (PG), STS-PROM, elective surgery, TF approach and PPM ≥ moderate were associated with *P *< 0.1 (Supplementary Table S4). After adjusting for confounders (see footnote in Table 3), the excess risk for all-cause mortality remained significant in the severe thinness (BMI <16 kg/m^2^) (adjusted hazard ratio [aHR], 3.00; 95% confidence interval [CI], 2.03–4.41, *P *< 0.001) and mild–moderate thinness (BMI 16–18.5 kg/m^2^) (aHR, 1.35; 95% CI, 1.03–1.76, *P *= 0.027) relative to the normal weight group (Table 3). In the multivariable analysis, age, diabetes mellitus, hemodialysis, peripheral artery disease, liver cirrhosis, NYHA, LVEF, STS-PROM, elective surgery, TF approach, and PPM ≥ moderate did not remain statistically significant (Supplementary Table S4).

**Table 3:** Risk analysis of BMI for 5-year follow-up mortality: Cox proportional hazards regression analysis

Variable	Univariable analysis		Multivariable analysis	
	Crude HR (95% CI)	*P*-value	Adjusted HR (95% CI)^a^	*P*-value
Severe thinness (BMI <16 kg/m^2^) (vs normal weight)	3.34 (1.98–5.64)	<0.001	3.00 (2.03–4.41)	**<0.001**
Mild–moderate thinness (BMI 16–18.5 kg/m^2^) (vs normal weight)	1.56 (1.14–2.12)	0.005	1.35 (1.03–1.76)	**0.027**

^a^ Adjusted with age, male sex, DM, eGFR, hemodialysis, albumin, hemoglobin, hypertension, COPD ≥ moderate, immunosuppressant drugs, peripheral artery disease, cerebrovascular disease, malignant disease, liver cirrhosis, NYHA≥III, atrial fibrillation, LVEF, mean PG, STS-PROM, elective surgery, TF-approach, PPM ≥ moderate.

Values in bold indicate statistically significant. BMI: body mass index; HR: hazard ratio; CI: confidence interval; DM: diabetes mellitus; eGFR: estimated glomerular filtration rate; COPD: chronic obstructive pulmonary disease; NYHA: New York Heart Association; LVEF: left ventricular ejection fraction; PG: pressure gradient; LV: left ventricular; STS-PROM: Society of Thoracic Surgeons Predicted Risk of Mortality; TF: transfemoral; PPM: prosthesis-patient mismatch.

## DISCUSSION

This study investigated the impact of severity of thinness on the clinical outcomes of patients with TAVI by comparing in-hospital and mid-term mortality among severe thinness, mild–moderate thinness and normal weight groups using data from a multicentre retrospective registry in Japan. The severe thinness group not only had a prolonged hospital stay but also an increased in-hospital mortality. In the mid-term period, there was an association between severity of thinness and cumulative mortality, with both cardiac and non-cardiac mortality increasing in the severe thinness group. Additionally, multivariable analysis demonstrated that severity of thinness was an independent risk factor for mortality after TAVI.

The preoperative risk scores significantly influence the treatment strategy for AS. Current risk scores, which are based on the patient databases in Western countries, cannot accurately calculate the surgical risk in underweight patients. This is because the average BMI of patients with TAVI in Western countries is 26–27 kg/m^2^, while very few patients have a BMI <20 kg/m^2^ [11]. This discrepancy between the predicted surgical risk score and actual patient risk can lead to unexpected complications and mortality. The number of patients undergoing TAVI is rapidly increasing globally [3, 4], not only in Western countries but also in East Asia [5]. The number of patients undergoing TAVI is expected to increase across several regions and countries. A notable characteristic of patients undergoing TAVI in East Asia is the high prevalence of underweight individuals [7]. Specifically, regions with large populations, such as Asia, India and Africa, have higher rates of underweight than obese individuals [12]. Therefore, the expansion of TAVI in these regions might increase the number of underweight patients who undergo TAVI. In patients with heart failure, mortality rates are increased by 2.32 folds in severe thinness and 1.31 in mild–moderate thinness compared with those in patients with normal weight [13]. Tezuka *et al.* [8] reported that in East Asian patients with TAVI, underweight status was associated with worse outcomes than normal-weight status. However, whether the severity of thinness in the underweight category is related to clinical outcomes remains unclear. This study provides important findings by demonstrating that the severity of thinness stratifies risk and that mortality is increased by 3.00 folds in severe thinness and 1.35 in mild–moderate thinness compared to that in patients with normal weight. When developing TAVI risk scores, it is crucial to create a scoring system that accurately assesses risk in underweight patients by incorporating the severity of thinness, particularly given the increase in the number of such patients.

Frailty is a condition with a poor prognosis characterized by reduced mobility and malnutrition. It is an independent risk factor for TAVI and can arise from various causes. Although there is no universally accepted definition for diagnosing frailty, being underweight is often included as a criterion [14]. In this study, albumin and hemoglobin levels decreased with increasing severity of thinness, indicating higher frailty rates and severity. Although this study did not identify the causes of thinness, the significantly higher rate of cardiac death in the severe thinness group suggested that some patients may have had cardiac cachexia. Furthermore, frailty is associated with an increased incidence of respiratory diseases [15], and the finding in this study that the incidence of respiratory diseases, including pneumonia, increased in proportion to the severity of thinness corroborates the influence of frailty. Preoperative nutritional therapy and rehabilitation for patients scheduled to undergo TAVI may prevent respiratory complications, shorten the postoperative hospitalization period and improve overall prognosis [16, 17]. The ongoing PERFORM-TAVR trial (NCT03522454) is of particular interest to determine whether preoperative nutritional therapy and rehabilitation interventions can improve outcomes in frail patients scheduled for TAVI.

Previous studies have suggested that the increased incidence of access complications due to narrow femoral arteries in underweight patients and the higher frequency of non-TF approaches, which may lead to increased bleeding complications, could be associated with poorer outcomes after TAVI [6, 11]. However, recent device advancements have made it possible to safely perform TAVI using the TF approach, even in patients with narrow-access femoral arteries. In this study, there were no significant differences in the rate of use of the TF approach among the three groups, and the choice of approach did not contribute to differences in outcomes. The implication of this study is that underweight patients undergoing TAVI are more likely to have poor prognosis because they may have cardiac cachexia and frailty. Even when treated safely with TF-TAVI, these patients remain at high risk for cardiac mortality, as well as non-cardiac mortality, including postoperative respiratory complications. Therefore, careful perioperative management and outpatient follow-up are crucial.

The study cohort included 13.1–23.1% patients with coronary artery disease (CAD). SAVR with coronary artery bypass grafting (CABG) was comparable to TAVI with percutaneous coronary intervention (PCI) in short-term outcomes [18], and in octogenarian patients, SAVR vs SAVR+CABG was reported to have comparable long-term outcomes [19, 20]. Therefore, a comparison of the long-term outcomes of SAVR+CABG and TAVI+PCI remains an important issue for future research. It should be noted that as highlighted by the present findings, high pacemaker implantation rate (8.0–15.0%) and PVL ≥ moderate (3.4–4.7%) still remain a concern in older patients undergoing TAVI. Additionally, the prevalence of CAD and malignant disease was lower in the severe thinness group, suggesting a potential bias. CAD was not a significant risk factor in the univariable analysis, whereas malignant disease was identified as an independent risk factor in the multivariable analysis. However, even after adjusting for malignant disease in the multivariable analysis, the severity of thinness remained an independent risk factor. And patients with severe thinness had a significantly higher prevalence of NYHA ≥ III, which may have influenced the outcomes; however, it was not an independent risk factor in the multivariable analysis.

Notably, in our study, while the severe thinness group had a smaller implanted prosthesis size, postoperative echocardiography showed a lower incidence of PPM without an increase in the mean PG than the other two groups. However, the mortality was significantly higher than that of the other groups. Although obesity patients generally have a better prognosis despite having a higher incidence of PPM after TAVI [21], the relationship between PPM and prognosis in underweight patients has not been well-demonstrated. Obesity patients may have a large body surface area (BSA), leading to underestimation of the effective orifice area index (EOAI), whereas underweight patients may have a small BSA, resulting in overestimation of the EOAI. Nevertheless, our results provide new insights that the severity of thinness may be a stronger risk factor than PPM.

This study has certain limitations. This was a retrospective multicenter observational study with a relatively small sample size; particularly, the sample size of severe thinness group was small. The multivariable logistic regression analysis did not converge owing to the very small number of in-hospital mortality events. There was a possibility that unmeasured confounding factors could have influenced the results. Specifically, frailty assessments including clinical frailty score and walking speed, and nutritional indicators (other than albumin), such as mid-upper arm circumference (MUAC), that can directly measure muscle and fat reserves, were not measured. Furthermore, data on changes in postoperative BMI were not collected. Improvements in hemodynamics after TAVI and postoperative nutritional status may influence BMI changes and subsequently affect prognosis. To validate this study and further mitigate bias, a large-scale propensity score-matched study or a prospective observational study incorporating quantitative assessments of frailty, detailed nutritional indicators such as MUAC, and the impact of postoperative BMI changes is needed.

## CONCLUSION

Severe thinness was associated with prolonged hospitalization and increased the incidence of in-hospital mortality in older patients (≥80 years) undergoing TAVI. Furthermore, severity of thinness was identified as an independent risk factor for mid-term mortality after TAVI. We need to recognize that accurate risk calculation in underweight patients is difficult, and careful patient selection is crucial. Improvements in the risk scoring are also required. Further a large-scale propensity score-matched study or prospective observational studies are required.

## Supplementary Material

ivaf120_Supplementary_Data

## Data Availability

The authors declare that all data in this article are available within the article.

## ABBREVIATIONS

aHR: Adjusted hazard ratio
AS: Aortic stenosis
BMI: Body mass index
CABG: Coronary artery bypass grafting
CAD: Coronary artery disease
CI: Confidence interval
LVEF: Left ventricular ejection fraction
NYHA: New York Heart Association
PPM: Prosthesis-patient mismatch
PVL: Paravalvular leak
SAVR: Surgical aortic valve replacement
STS-PROM: Society of Thoracic Surgeons Predicted Risk of Mortality
TAVI: Transcatheter aortic valve implantation
TF: Transfemoral
