# Supplementary material for: Severity of thinness amplifies mortality risk in patients with transcatheter aortic valve implantation
Source: Interdiscip Cardiovasc Thorac Surg. 2025 Jun 20;40(6):ivaf120. doi: 10.1093/icvts/ivaf120 (PMC12202768; doi:10.1093/icvts/ivaf120)
Supplement: ivaf120_Supplementary_Data [file ivaf120_supplementary_data.zip › Supplementary_files_for_Revision_250617.docx]

**Supplementary Files**

**Severity of Thinness Amplifies Mortality Risk in Patients with Transcatheter Aortic Valve Replacement**

**Table S1. Detailed cause of in-hospital mortality**

| **Variable** | | **Severe thinness**  **(n = 4/60 deaths [6.7%])** | | **Mild-moderate thinness**  **(n = 0/213 deaths [0.0%])** | | | **Normal weight**  **(n = 8/1161 deaths [0.7%])** | |
| --- | --- | --- | --- | --- | --- | --- | --- | --- |
| Cardiac mortality | | 1 | (25.0) | | 0 | (0.0) | 3 | (37.5) |
|  | Heart failure | 1 | (25.0) | | 0 | (0.0) | 3 | (37.5) |
| Non-cardiac mortality | | 3 | (75.0) | | 0 | (0.0) | 5 | (62.5) |
|  | Pneumonia | 2 | (50.0) | | 0 | (0.0) | 0 | (0.0) |
|  | Respiratory | 0 | (0.0) | | 0 | (0.0) | 1 | (12.5) |
|  | Gastrointestinal | 1 | (25.0) | | 0 | (0.0) | 1 | (12.5) |
|  | Urinary tract infection | 0 | (0.0) | | 0 | (0.0) | 2 | (25.0) |
|  | Stroke | 0 | (0.0) | | 0 | (0.0) | 1 | (12.5) |
| Data are expressed as n (%). | | | | | | | | |

**Table S2. One-year follow-up echocardiography**

| **Variable** | | **Severe thinness**  **(n = 26)** | **Mild-moderate thinness**  **(n = 115)** | **Normal weight**  **(n = 598)** | ***P* value** |
| --- | --- | --- | --- | --- | --- |
|  | LVEF (%) | 69.5 [61.8–72.3] | 66.0 [61.0–72.0] | 67.3 [62.0–73.0] | 0.188 |
|  | LV mass index (g/m2) | 90.8 [79.9–116.1] | 99.2 [79.0–120.1] | 101.0 [86.4–119.9] | 0.192 |
|  | mean PG (mmHg) | 7.5 [4.2–10.8] | 8.0 [6.0–11.0] | 9.0 [6.0–13.0] | **0.039** |
|  | PPM ≥ moderate | 2 (6.3) | 16 (10.5) | 133 (14.6) | 0.174 |
|  | PVL grade ≥ mild | 18 (69.2) | 74 (64.4) | 421 (69.4) | 0.435 |
| Data are expressed as n (%) or median (interquartile range).  Values in bold indicate statistically significant.  LVEF, left ventricular ejection fraction; LV, left ventricular; PG, pressure gradient; PPM, prosthesis-patient mismatch; PVL, paravalvular leak. | | | | | |

**Table S3. Detailed cause of midterm mortality**

| **Variable** | | **Severe thinness (n = 32/60 deaths [53.3%])** | | | **Mild-moderate thinness (n = 74/213 deaths [34.7%])** | | **Normal weight (n = 296/1161 deaths [25.5%])** | |
| --- | --- | --- | --- | --- | --- | --- | --- | --- |
| Cardiac mortality | | 12 | (37.5) | 32 | | (43.2) | 118 | (39.9) |
|  | Heart failure | 6 | (18.8) | 13 | | (17.6) | 45 | (15.2) |
|  | Unknown | 5 | (15.6) | 16 | | (21.6) | 66 | (22.3) |
|  | Sudden death | 1 | (3.1) | 1 | | (1.4) | 4 | (1.4) |
|  | MI | 0 | (0.0) | 2 | | (2.7) | 3 | (1.0) |
| Non-cardiac mortality | | 20 | (62.5) | 42 | | (56.8) | 178 | (60.1) |
|  | Pneumonia | 7 | (21.9) | 12 | | (16.2) | 36 | (12.2) |
|  | Respiratory | 3 | (9.4) | 3 | | (4.1) | 13 | (4.4) |
|  | GI | 5 | (15.6) | 2 | | (2.7) | 9 | (2.7) |
|  | Cancer | 2 | (6.3) | 8 | | (10.8) | 27 | (9.1) |
|  | Senility | 1 | (3.1) | 11 | | (14.9) | 16 | (5.4) |
|  | UTI | 1 | (3.1) | 4 | | (5.4) | 26 | (8.8) |
|  | Stroke | 0 | (0.0) | 1 | | (1.4) | 24 | (8.1) |
|  | Renal failure | 0 | (0.0) | 0 | | (0.0) | 8 | (2.7) |
|  | Others | 1 | (3.1) | 1 | | (1.4) | 19 | (6.4) |
| Data are expressed as n (%). MI, myocardial infarction; GI, gastrointestinal; UTI, urinary tract infection. | | | | | | | | |

**Table S4. Risk analysis of BMI for five-year follow-up mortality: Cox proportional hazards regression univariable and multivariable analysis**

| **Variable** | **Univariable analysis** | |  | **Multivariable analysis** | |
| --- | --- | --- | --- | --- | --- |
|  | **Crude HR (95% CI)** | ***P* value** |  | **Adjusted HR (95% CI)** | ***P* value** |
| Severe thinness (BMI <16 kg/m^2^)  (vs. normal weight) | 3.34 (1.98–5.64) | **<0.001** |  | 3.00 (2.03–4.41) | **<0.001** |
| Mild-moderate thinness (BMI 16–18.5 kg/m^2^)  (vs. normal weight) | 1.56 (1.14–2.12) | **0.005** |  | 1.35 (1.03–1.76) | **0.027** |
| Age | 1.01 (0.99–1.03) | 0.269 |  | 1.01 (0.99–1.04) | 0.057 |
| Male sex | 1.49 (1.22–1.82) | **<0.001** |  | 1.43 (1.16–1.77) | **<0.001** |
| DM | 1.21 (0.97–1.50) | 0.079 |  | 1.20 (0.95–1.50) | 0.127 |
| eGFR (mL/min/1.73 m^2^) | 0.98 (0.98–0.99) | **<0.001** |  | 0.99 (0.98–0.99) | **<0.001** |
| Hemodialysis | 2.67 (1.95–3.66) | **<0.001** |  | 1.33 (0.86–2.06) | 0.191 |
| Albumin (g/dL) | 0.45 (0.38–0.55) | **<0.001** |  | 0.59 (0.48–0.74) | **<0.001** |
| Hemoglobin (g/dL) | 0.83 (0.78–0.89) | **<0.001** |  | 0.92 (0.86–0.98) | **0.012** |
| Hypertension | 1.48 (1.20–1.82) | **<0.001** |  | 1.41 (1.13–1.76) | **0.002** |
| COPD ≥ moderate | 1.76 (1.32–2.34) | **<0.001** |  | 1.42 (1.05–1.92) | **0.023** |
| Immunosuppressant drugs | 1.36 (1.00–1.84) | **0.048** |  | 1.49 (1.08–2.06) | **0.015** |
| Peripheral artery disease | 1.49 (1.12–1.98) | **0.006** |  | 1.14 (0.84–1.54) | 0.412 |
| Cerebrovascular disease | 1.59 (1.22–2.07) | **<0.001** |  | 1.43 (1.09–1.88) | **0.011** |
| Malignant disease | 1.75 (1.28–2.38) | **<0.001** |  | 1.75 (1.26–2.46) | **0.001** |
| Liver cirrhosis (Child ≥B) | 2.98 (1.59–5.59) | **<0.001** |  | 1.64 (0.81–3.34) | 0.168 |
| Coronary artery disease | 1.13 (0.90–1.41) | 0.286 |  |  |  |
| NYHA ≥III | 1.66 (1.36–2.03) | **<0.001** |  | 1.21 (0.97–1.52) | 0.093 |
| Atrial fibrillation | 2.20 (1.76–2.75) | **<0.001** |  | 1.44 (1.13–1.85) | **0.004** |
| LVEF (%) | 0.99 (0.98–0.99) | **<0.001** |  | 1.01 (0.99–1.01) | 0.135 |
| Mean PG (mmHg) | 0.98 (0.97–0.99) | **<0.001** |  | 0.99 (0.98–0.99) | **0.003** |
| LV mass index (g/m^2^) | 1.00 (0.99–1.00) | 0.889 |  |  |  |
| STS-PROM (%) | 1.08 (1.06–1.10) | **<0.001** |  | 1.02 (0.99–1.05) | 0.095 |
| Elective | 0.51 (0.34–0.77) | **0.001** |  | 0.87 (0.54–1.42) | 0.583 |
| TF-approach | 0.66 (0.45–0.96) | **0.029** |  | 0.98 (0.66–1.45) | 0.921 |
| PPM ≥ moderate | 1.35 (0.53–1.02) | 0.067 |  | 1.41 (0.46–1.09) | 0.114 |
| PVL ≥ moderate | 1.15 (0.68–1.93) | 0.596 |  |  |  |
| PVL ≥ mild | 1.03 (0.82–1.29) | 0.815 |  |  |  |
| Values in bold indicate statistically significant.  BMI, body mass index; HR, hazard ratio**;** CI, confidence interval; DM, diabetes mellitus; eGFR, estimated glomerular filtration rate; COPD, chronic obstructive pulmonary disease; NYHA, New York Heart Association; LVEF, left ventricular ejection fraction; PG, pressure gradient; LV, left ventricular; STS-PROM, Society of Thoracic Surgeons Predicted Risk of Mortality; TF, transfemoral; PPM, prosthesis-patient mismatch; PVL, paravalvular leak. | | | | | |

**Figure S1. Kaplan-Meier curves for the cumulative incidence of all-cause mortality (A), cardiac mortality (B), and non-cardiac mortality (C) in the underweight group (BMI < 18.5) vs. the normal weight group (BMI ≥ 18.5).**

BMI, body mass index

**Figure S2. Rate of cardiac and non-cardiac mortality during the follow-up**

BMI, body mass index

**Figure S3. Kaplan-Meier curves with censoring for the cumulative incidence of all-cause mortality (A), cardiac mortality (B), and non-cardiac mortality (C) in the total cohort**

BMI, body mass index

**Figure S4. Kaplan-Meier curves for the cumulative incidence of all-cause mortality (A), cardiac mortality (B), and non-cardiac mortality (C) in the cohort excluding patients with liver cirrhosis**

BMI, body mass index
